# Does sexuality matter? A cross-sectional study of drug use, social injecting, and access to injection-specific care among men who inject drugs in Melbourne, Australia

**DOI:** 10.1186/s12954-023-00737-6

**Published:** 2023-01-23

**Authors:** Sophia E. Schroeder, A. L. Wilkinson, D. O’Keefe, A. Bourne, J. S. Doyle, M. Hellard, P. Dietze, A. Pedrana

**Affiliations:** 1 Disease Elimination Program, Burnet Institute, 85 Commercial Road, Melbourne, 3004 Australia; 2 School of Public Health and Preventive Medicine, Monash University, Melbourne, Australia; 3 Australian Research Centre for Sex, Health and Society, La Trobe University, Bundoora, Australia; 4 Kirby Institute, UNSW Sydney, Sydney, Australia; 5 Department of Infectious Diseases, The Alfred Hospital and Monash University, Melbourne, Australia; 6 Doherty Institute and Melbourne School of Population and Global Health, University of Melbourne, Melbourne, Australia; 7 National Drug Research Institute, Curtin University, Perth, Australia

**Keywords:** Sexual minority men, Injection drug use, People who inject drugs, Injecting practices, Sexuality and sociability

## Abstract

**Background:**

Gay, bisexual and other men who have sex with men (GBMSM) are overrepresented in cohorts of people who inject drugs. GBMSM’s substance use is usually explored in the context of its contribution to sexual risk. We examined drug use practices, connectedness to other people who inject drugs, peer-to-peer injecting, and access to care among men who inject drugs in Melbourne, Australia. We aim to describe similarities and differences in these parameters for GBMSM and other men.

**Methods:**

Data were drawn from a prospective cohort study of people who inject drugs conducted in Melbourne, Australia, since 2009. This cross-sectional study used data collected between 2016 and 2021. Descriptive statistics were used to assess differences between GBMSM and other men.

**Results:**

Of 525 men who injected drugs over the study period, 48 (9%) identified as gay or bisexual, or reported sex with other men in the past 12 months. GBMSM and other men reported similar socio-demographics, drug practices (age of injecting initiation, most injected drug, peer-to-peer injecting, receptive syringe sharing) and access to injecting-specific care (drug treatment, source of needle-syringes). A significantly greater percentage of GBMSM reported past 12-month hepatitis C testing (69% vs. 52%, *p* = 0.028) and preferring methamphetamine (31% vs. 16%, *p* = 0.022). A higher percentage of GBMSM reported knowing > 50 other people who inject drugs (46% vs. 37%), but this difference was not statistically significant. Both groups primarily obtained injecting equipment from needle-syringe programs; a minority had accessed injecting-specific primary care.

**Conclusion:**

Men who injected drugs in this cohort and those who identified as GBMSM reported similar drug and health-seeking practices. The higher prevalence of methamphetamine injecting among GBMSM may warrant different harm reduction support for this group. Health promotion should utilise opportunities to connect men who inject drugs in Melbourne to injecting-specific primary health care.

## Background

Injecting drug use is a major contributor to drug-related disease burden worldwide [1] and a heavily stigmatised social practice [2]. People who inject drugs typically have higher rates of morbidity and mortality than the broader population, due largely to drug overdose, impacted mental health, and transmissible infections such as HIV and hepatitis C virus (HCV) [3]. They are more likely to experience unstable housing or homelessness; be vulnerable to police arrest and incarceration; and to engage in sex work—all of which are associated with increased risk of negative health sequelae [4–6]. This experience of social and health inequity means that people who inject drugs are a focal point for public health interventions [7].

In Australia [8, 9] and internationally, gay, bisexual and other men who have sex with men (GBMSM) are more likely to report illicit and injecting substance use than the general population [9–16] and are often overrepresented in studies of people who inject drugs [17, 18]. Most studies show that crystal methamphetamine is the drug GBMSM report injecting most often [9, 17–19], including in the context of sexual activity [20–24].

The intersection of injection and sexual minority practices is associated with distinct risks and potential harms [25–28]. Studies comparing the characteristics of GBMSM who inject drugs and other people who inject drugs have found GBMSM have a higher risk of intentional overdose [29], a lower likelihood of accessing addiction treatment [18] and heightened ‘sexual risk’ (typically implying condomless anal intercourse and greater number of sexual partners) [30]. Within samples of GBMSM, those who report injecting drug use experience greater socio-structural adversities, including economic disadvantage, homelessness, criminalisation, stigma and violent victimisation [19, 31, 32]*.* Evidence suggests that GBMSM who inject drugs are more likely to occupy distinct sociocultural spaces than other people who inject drugs [17], with qualitative analyses highlighting GBMSM-specific sociocultural meanings of injecting drug use [33, 34]. These include gaining access to particular socio-sexual networks and establishing relationships with other men [26, 27], where peer-to-peer injecting (i.e. injecting each other) can foster greater intimacy and social capital [25, 35].

The evidence above underpins the suggestion that GBMSM who inject drugs are distinct from other people who inject drugs [17], including in terms of their socio-demographic characteristics, drug practices, and access to health care. We sought to investigate this possibility by assessing similarities and differences between GBMSM and other men in a cohort of people recruited based on their engagement in injecting drug use in Melbourne, Australia. Specifically, we aimed to explore if the findings from the literature highlighted above as being important for understanding GBMSM’s drug use (i.e. methamphetamine use, engagement in social networks featuring injection drug use [27], and peer-to peer injecting [34]) translate into quantifiable differences, including in accessing of injecting-specific health care.

## Methods

### Study design and setting

We used data from the Melbourne Injecting Drug User Cohort Study (SuperMIX). SuperMIX is a longitudinal cohort study established in 2008 to identify trajectories of injecting drug use among people who inject drugs in Melbourne, Australia. Recruitment and data collection procedures have been described in detail previously [36, 37]. Briefly, participants were recruited in and around known drug markets in metropolitan Melbourne, Australia, through a combination of respondent-driven sampling, snowball sampling and street outreach methods. Two main recruitment waves occurred 2008–2010 and 2017–2018, but recruitment to replace those lost-to-follow-up occurs continuously. SuperMIX eligibility criteria include being aged ≥ 18 years and having injected either heroin or amphetamines at least monthly in the six months preceding enrolment. At baseline and annual follow-up visits, participants complete interviewer-administered questionnaires collecting information on socio-demographics, drug practices, service utilisation, health and well-being measures, and HIV and HCV testing history (self-report). Differences between baseline and follow-up questionnaires are minimal, except that the baseline questionnaire contains a greater proportion of time-invariant factors (e.g. country of birth) and lifetime recall periods (i.e. ‘ever’), while follow-up questionnaires inquire about time-varying factors (e.g. drug practices) ‘since last seen’. Participants are remunerated AUD30 for their time and expertise at each encounter.

### Participants

We created a cross-sectional sample from cohort participants’ most recent survey (baseline or follow-up) among those who completed at least one questionnaire between August 2016 and August 2021 (*N* = 1016). From this, we created a study sample consisting of cisgender men who had recently injected drugs and could be categorised as either GBMSM or non-GBSM (*n* = 525). We included participants who:

- Answered ‘male’ to both questions: *‘What was your sex assigned at birth?’* (male, female) and *‘Which gender identity best describes you?’* (male, female, non-binary/gender-fluid, other); AND
- Gave a valid response to the question: *“What illicit or non-prescribed drug did you inject most during the last month?;* AND
- Gave a valid response to the question: *‘What is your sexual orientation?* and/or reported ≥ 1 male sexual partner in the previous 12 months.

### Measures

We categorised participants into two groups. Those who reported gay, bisexual or queer sexual orientation and/or who reported any male sexual partners in the previous 12 months were categorised as GBMSM; the remaining men were categorised as non-GBMSM.

#### Socio-demographics

We describe the following socio-demographic characteristics by GBMSM status: age at interview (years, continuous); country of birth (Australia, other); identification as either Aboriginal, Torres Strait Islander, or both (yes, no); educational attainment (< year 10, year 10–12, tertiary/diploma/trade, other); average weekly income (< $400, $400–$999, ≥ $1000); primary income source (wage or salary, government support, other); living circumstances (alone, with others) and housing stability (stable, unstable/homeless). Unstable housing was defined as living in a boarding house/hostel, shelter/refuge or caravan park, ‘couch surfing’ or ‘sleeping rough’ [37, 38].

#### Self-reported HIV and HCV testing and status

Participants were asked if they had *‘ever’* tested for HCV and HIV at their baseline interview, and if they had tested *‘in the past 12 months/since last seen*’ at subsequent interviews. Those who responded ‘yes’ were then asked how long ago this test had taken place. From this we derived past 12 month HCV testing (yes, no) and past 12 month HIV testing (yes, no).

We report self-reported test results among those responding *‘yes’* to having tested in the past 12 months.

#### Injecting history and practice

To understand differences in overall drug preferences, we derived ‘drug of choice’ from the question *‘What’s your favourite illicit drug of choice?’* and categorised responses into heroin, meth/amphetamine, or other. To understand differences in recent use of drugs, we derived ‘drug most injected in the previous month’, categorised as heroin, meth/amphetamine, or other. Number of social contacts who also inject drugs was derived from the question: *‘How many people do you know who inject drugs?’* (continuous), categorised into 0, 1–20, 21–50, and > 50. Numbers of people injected with in the past six month (continuous) and in the past four weeks (continuous) were derived from the questions *‘During the past six months/past one month, how many different people have you injected with, in the same time and place?’* and categorised into 0, 1, 2–5 and ≥ 6. Performing injecting assistance (yes, no) and receiving injecting assistance (yes, no) were derived from the questions: *‘In the last month, how many times have you assisted/performed another person’s injection?’* and ‘*In the last month, how many times has another person assisted/performed your injection for you*?’ Receptive syringe sharing was derived from the question: *‘In the last month, how many times have you injected with another person’s used syringe?’*, and categorised into 0, 1–2, 3–5, and ≥ 6.

#### Access to care

Participants were asked if they had *‘ever’* accessed any form of drug treatment at their baseline interview, and if they had accessed drug treatment *‘in the past 12 months/since last seen*’ at subsequent interviews (yes, no). We report on ‘ever’ accessing any form of drug treatment among participants who only completed a baseline survey, and accessing drug treatment *‘in the past 12 months/since last seen*’ among participants who completed a follow-up survey. Participants who responded ‘yes’ to having accessed treatment were asked if they were currently on drug treatment, including: methadone, suboxone, buprenorphine, Sublocade, Buvidal, naltrexone, drug counselling, and self-help groups. From this, we derived *‘currently on any drug treatment’* (yes, no). Main source of needles and syringes in the past month (categorical) was dichotomised into needle-syringe program (NSP) vs. other. Participants were asked if they had visited injecting-specific primary care clinics for reasons other than accessing the co-located NSP (yes, no).

### Statistical methods

Descriptive statistics were generated for demographics and drug use practices variables stratified by GBMSM and non-GBMSM. We tested for differences between groups using Pearson’s χ2 or Fisher’s exact tests, and tested for differences in means of continuous variables (Welch’s t test, after checking for normal distribution). All tests were two-sided, and we set a statistical significance level of *α* = 0.05. Data management and analyses were conducted using Stata 15.1 (StataCorp, College Station, Texas).

### Treatment of missing data

Participants with missing data on variables of interest were excluded from test of differences between groups. We report the percentage missing for each variable below, except when cell sizes < 5 as per our ethical requirements.

## Results

### Study sample

**Fig. 1:**
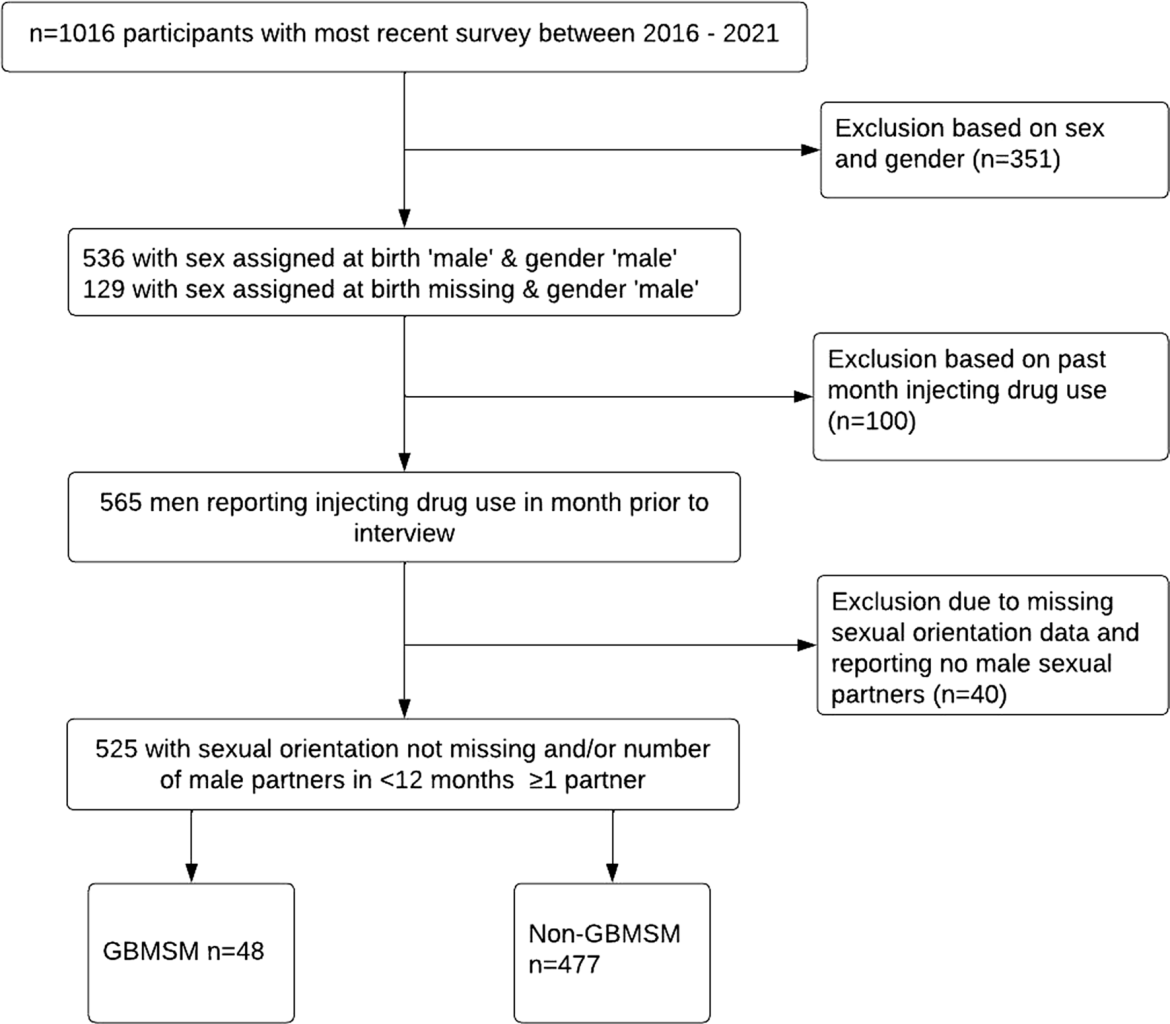
Flowchart of study sample selection

A total of 1,016 participants completed at least one interview between 01 January 2016 and 31 December 2021 inclusive. We excluded 351 participants (35%) who reported ‘female’ sex at birth, and/or reported ‘female’ or ‘other’ gender. Of 665 men, 100 (15%) were excluded because of missing data on past-month injecting drug use. A further 40 (6%) were excluded because of missing data on sexual orientation and reporting zero male sexual partners since their last study visit (< 12 months). The final study sample consisted of 525 men (Fig. 1).

#### Demographic data

Of 525 included men, 48 (9%) reported gay, bisexual, or queer orientation and/or having had sex with men in the previous 12 months. Of these, 8 (17%) identified as gay and 28 (58%) as bisexual. The remaining 24% reported their sexual orientation as heterosexual, queer or ‘other’, or were missing data on this question, and they reported ≥ 1 male sexual partners over the past 12 months. There was no difference between groups regarding the distribution of baseline versus follow-up interviews, with 31 (65%) GBMSM and 335 (70%) non-GBMSM contributing data from a follow-up survey (*p* = 0.417).In our sample, GBMSM were significantly younger than non-GBMSM (38 years vs. 40 years, *p* = 0.049). GBMSM and non-GBMSM were comparable across the remaining socio-demographic variables examined, with no significant differences identified (Table 1).

**Table 1:** Socio-demographics, HCV and HIV testing and self-report status among men who inject drugs in Melbourne, Australia, August 2016–August 2021, stratified by GBMSM status (*N* = 525)

Characteristic	Total	GBMSM	Non- GBMSM	*p-*value
	*N* = 525	*n* = 48	*n* = 477	
	*N* (%)	*N* (%)	*N* (%)	
*Interview completion year*				
2016	< 5 (< 5)	< 5	0 (0.0)	**0.007**^**++**^
2017	26 (5.0)	5 (10.4)	21 (4.4)	
2018	147 (28.0)	14 (29.2)	133 (27.9)	
2019	172 (32.8)	10 (20.8)	162 (34.0)	
2020	151 (28.8)	15 (31.3)	136 (28.5)	
2021	27 (5.14)	< 5 (< 5)	25 (5.2)	
Mean age at interview (years, SD), (*n* = 524)	39.8 (8.2)	37.5 (8.4)	40 (8.1)	0.049*
*Country of birth*				
Australia	446 (85.0)	42 (87.5)	404 (84.7)	0.391^+^
Other	78 (14.9)	5 (10.4)	73 (15.3)	
Missing	< 5 (< 5)	< 5 (< 5)	0 (0.0)	
*Aboriginal or Torres strait islander identity*				
Yes	87 (16.6)	11 (22.9)	76 (15.9)	0.220^+^
No	436 (83.1)	37 (77.1)	399 (83.7)	
Missing	< 5 (< 5)	0 (0.0)	< 5 (< 5)	
*Educational attainment*				
< Year 10	159 (30.3)	11 (22.9)	148 (31.0)	0.439^+^
Year 10–12	217 (41.3)	19 (39.6)	198 (41.5)	
Tertiary/diploma/trade	104 (19.8)	13 (27.1)	91 (19.1)	
Other	42 (8.2)	5 (10.4)	38 (8.0)	
Missing	< 5 (< 5)	0 (0)	< 5 (< 5)	
*Average weekly income*				
< $400	239 (45.5)	15 (31.3)	224 (47.0)	0.097^+^
$400–$999	244 (46.5)	28 (58.3)	216 (45.3)	
> $1000	38 (7.2)	5 (10.4)	33 (6.9)	
Missing	< 5 (< 5)	0 (0)	< 5 (< 5)	
*Main source of income*				
Wage or Salary	42 (8.0)	5 (10.4)	37 (7.8)	0.516^++^
Government pension or benefits	437 (83.24)	38 (79.2)	399 (83.7)	
Other	40 (7.6)	5 (10.4)	35 (7.3)	
Missing	6 (1.1)	0 (0.0)	6 (1.3)	
*Living circumstances*				
Alone	295 (56.2)	29 (60.4)	266 (55.8)	0.596^+^
With others	213 (40.6)	18 (37.5)	195 (40.9)	
Missing	17 (3.3)	< 5 (< 5)	16 (3.3)	
*Housing stability*				
Stable	243 (46.3)	23 (47.9)	220 (46.1)	0.822^+^
Unstable/Homeless	281 (53.5)	25 (52.1)	257 (53.7)	
Missing	< 5 (< 5)	0 (0.0)	< 5 (< 5)	
*HCV tested*^*a*^* in past 12 months*				
Yes	282 (53.7)	33 (68.8)	249 (52.2)	**0.028**^**+**^
No	218 (41.5)	13 (27.1)	205 (43.0)	
Missing	25 (4.8)	< 5 (< 5)	23 (4.8)	
*HCV status (self-report), among those tested in the past 12 months (n = 282)*				
Positive (Ab positive, PCR positive)	81 (28.7)	10 (30.3)	71 (28.5)	0.407^+^
Negative (Ab negative, PCR negative)	60 (21.3)	9 (27.3)	51 (20.5)	
Exposed/negative (Ab positive, PCR negative)	117 (41.5)	10 (30.3)	107 (43.0)	
Missing	24 (8.5)	< 5 (< 5)	20 (8.0)	
*HIV tested in past 12 months*				
Yes	274 (52.2)	29 (60.4)	245 (51.4)	0.278^++^
No	220 (41.9)	17 (35.4)	203 (42.6)	
Missing	31 (5.9)	< 5 (< 5)	29 (6.1)	

Bold indicates highlight* p*-values indicating statistically significant differences between the compared groups

All cells of < 5 have been suppressed. *p*‐values were derived using ^+^Pearson’s χ2 test or, alternatively, ^++^Fisher’s exact test on non-missing data when expected cell counts were ≤ 5 for categorical variables, and *Welch’s *t* test for continuous variables

^a^Any hepatitis C test

*Ab* Antibody; *HCV* *Hepatitis C virus*; *neg* *negative*; *PCR* Polymerase chain reaction

#### HCV and HIV testing

A significantly greater percentage of GBMSM compared to non-GBMSM reported past 12-month HCV testing (68% vs. 52%, respectively, *p* = 0.028). Of those tested, similar percentages of GBMSM and non-GBMSM reported testing positive for HCV infection (antibody positive and PCR positive, 30% vs. 29%, respectively), testing negative (antibody negative, PCR negative; 27% vs. 20%, respectively), and past exposure to HCV but no active infection (antibody positive, PCR negative; 30% vs. 43%, respectively).

There was no significant difference in the distribution of GBMSM and non-GBMSM reporting past 12-month HIV testing (60% vs. 51%, respectively, *p* = 0.278). Among those tested, overall < 5 participants returned a positive test result, with no significant differences between groups regarding HIV result (*p* = 0.179, data not shown in Table 1).

#### Drug use

On average, participants were 18 years old when they first injected drugs (Table 2). For GBMSM, the earliest and latest age of initiation was 13 and 26 years, and 8 and 41 years among non-GBMSM (data not shown).

**Table 2:** Drug use patterns and social injecting among men who inject drugs by GBMSM status, in Melbourne, Australia, August 2016–August 2021 (*N* = 525)

Characteristic	Total	GBMSM	Non-GBMSM	*p*-value
	*N* = 525	*N* = 48	*N* = 477	
	*N* (%)	*N* (%)	*N* (%)	
*Drug use factors*				
Age of injection initiation (years), Mean (SD)	18 (5)	17 (3)	18 (5)	0.267*
Main drug of choice				
Heroin^a^	344 (65.5)	28 (58.3)	316 (66.3)	**0.022**^**+**^
Meth/amphetamines	90 (17.14)	15 (31.3)	75 (15.7)	
Other^b^	86 (16.4)	5 (10.4)	81 (17.16)	
Missing	5 (0.95)	0 (0.0)	5 (1.1)	
Drug injected most in month prior to interview				
Heroin^c^	268 (70.1)	29 (60.4)	339 (71.1)	0.098^+^
Meth/amphetamines	145 (27.6)	19 (39.6)	126 (26.4)	
Other^d^	12 (2.3)	0 (0.0)	12 (2.5)	
Missing	0 (0.0)	0 (0.0)	0 (0.0)	
*Social injecting practices*				
Number of social contacts who inject				
Zero	6 (1.1)	0 (0.0)	6 (1.3)	0.811^++^
1–20	149 (28.4)	12 (25.0)	137 (28.7)	
21–50	159 (30.3)	14 (29.2)	145 (30.4)	
> 50	200 (38.1)	22 (45.8)	178 (37.3)	
Missing	11 (2.1)	0 (0.0)	11 (2.3)	
Number of people injected with in the past six months				
Zero	73 (13.9)	7 (14.6)	66 (13.8)	0.963^+^
1–2 person	145 (27.6)	15 (31.3)	130 (27.3)	
3–5 people	129 (24.6)	11 (22.9)	118 (24.7)	
> 6 people	164 (31.2)	15 (31.3)	149 (31.2)	
Missing	14 (2.7)	0 (0.0)	14 (2.9)	
Number of people injected with in past month				
Zero	113 (21.5)	14 (29.2)	99 (20.8)	0.527^+^
1–2 person	178 (33.9)	14 (29.2)	164 (34.4)	
3–5 persons	127 (24.2)	10 (10.8)	117 (24.5)	
> 6 persons	93 (17.7)	10 (10.8)	83 (17.4)	
Missing	14 (2.7)	0 (0.0)	14 (2.9)	
Provided injecting assistance in past month				
No	301 (57.3)	29 (60.4)	272 (57.0)	0.477^+^
Yes	217 (41.3)	17 (35.4)	200 (41.9)	
Missing	7 (1.3)	< 5 (< 5)	5 (1.1)	
Received injecting assistance in past month				
No	443 (84.4)	40 (83.3)	403 (84.5)	0.772^+^
Yes	75 (14.3)	6 (12.5)	69 (14.5)	
Missing	7 (1.3)	< 5 (< 5)	5 (1.1)	
Times reused another person’s needle/syringe in past month				
Zero	463 (88.2)	42 (87.5)	421 (88.3)	0.531^++^
1–2 times	36 (6.9)	5 (10.4)	31 (6.5)	
3–5 times	10 (1.9)	0 (0.0)	10 (2.1)	
> 6 times	8 (1.5)	< 5 (< 5)	7 (1.5)	
Missing	8 (1.5)	0 (0.0)	8 (1.7)	
*Health care access*				
Ever accessed any drug treatment, among those who completed a baseline survey only (*n* = 159)				
No	27 (17.0)	< 5 (< 25)	25 (17.6)	0.739^++^
Yes	132 (83.0)	15 (88.2)	117 (82.4)	
Missing	0 (0.0)	0 (0.0)	0 (0.0)	
Accessed any drug treatment in past 12 months/since last seen, among those who completed a follow-up survey (*n* = 366)				
No	116 (31.7)	7 (22.6)	109 (32.5)	0.250^+^
Yes	249 (68.0)	24 (77.4)	225 (67.2)	
Missing	< 5 (< 5)	0 (0.0)	< 5 (< 5)	
Currently on any drug treatment^f^				
No	268 (51)	27 (56.3)	241 (50.5)	0.449^+^
Yes	257 (49.0)	21 (43.8)	236 (49.5)	
Missing	0 (0.0)	0 (0.0)	0 (0.0)	
Main source of needle/syringes in past month				
NSP	399 (76.0)	37 (77.1)	362 (75.9)	0.891^+^
Other^e^	82 (15.6)	8 (16.7)	74 (15.5)	
Missing	44 (8.4)	< 5 (< 5)	41 (8.6)	
Access of IDU-specific primary care clinic for reasons other than NSP in past 12-months				
No	376 (71.6)	37 (77.1)	339 (71.1)	0.401^+^
Yes	147 (28.0)	11 (22.9)	136 (28.5)	
Missing	< 5 (< 5)	0 (0.0)	< 5 (< 5)	

Bold indicates highlight* p*-values indicating statistically significant differences between the compared groups

All cells of < 5 have been suppressed. *p*‐values were derived using on ^+^Pearson's χ2 test or, alternatively, ^++^Fisher's exact test on non-missing data when expected cell counts were ≤ 5 for categorical variables, and *Mann–Whitney U test (t test) for continuous variables,

^a^Category includes participants who specified equal preference for heroin and methamphetamine in freetext (*n* = 1 non-GBMSM)

^b^Category includes participants who specified cannabis, methadone, suboxone, ‘other’ opiates (e.g. codeine), benzodiazepines, cocaine, ecstasy/MDMA, hallucinogens, and those who specified in freetext fentanyl (*n* = 2), dexamphetamine (*n* = 1), homebake (*n* = 1), synthetic cannabis (*n* = 1) and ‘no drug’ (*n* = 3) (all non-GBMSM)

^c^Category includes participants who specified combined heroin and methamphetamine injecting in freetext (*n* = 2 non-GBMSM), and combining heroin and unisom in freetext (*n* = 1 GBMSM)

^d^Category includes participants who specified cocaine, morphine, buprenorphine, suboxone, cannabis; or who selected ‘other’ drug most injected, specifying ‘homebake’ in freetext (*n* = 1 non-GBMSM)

^e^Category includes participants who specified partner/friend, dealer, chemist/pharmacy, syringe vending machine, mobile outreach NSP van, supervised injecting facility, other

^f^Drug treatment includes Methadone, Suboxone, Buprenorphine, Sublocade, Buvidal, Naltrexone, Drug counselling, Self-help group (e.g. NA, AA, and SMART recovery)

*IDU* Injecting drug use; *NSP* Needle-syringe program

A majority of both GBMSM and non-GBMSM reported heroin as their main drug of choice and drug injected most in the month prior to interview. However, a significantly greater percentage of GBMSM compared to non-GBMSM reported meth/amphetamines as their main drug of choice (31% vs. 16%, respectively, *p* = 0.022). A higher percentage of GBMSM compared to non-GBMSM, though not significantly different, also reported methamphetamine as their most injected drug (40% vs. 26%, respectively, *p* = 0.098).

#### Social injecting practices

There was no statistically significant difference between GBMSM and non-GBMSM groups in the distribution of knowing > 50 other people who inject drugs (46% and 37%, respectively, *p* = 0.527). All GBMSM reported knowing at least one other person who injects drugs, while 1% of non-GBMSM reported knowing none.

Across both groups, the percentage of participants injecting with a greater number of people increased as the recall period increased, more so among GBMSM. In the past one month, 14 GBMSM (29%) reported always injecting alone, or injecting with 1–2 other people (compared to 99 (21%) and 164 (35%) of non-GBMSM, respectively)). Ten GBMSM (11%) reported injecting with 2–5 and ≥ 6 people (vs. 117 (25%) and 83 (17%) of non-GBMSM, respectively, *p* = 0.527). Over six months, similar proportions of GBMSM and non-GBMSM reported injecting alone (15% vs. 14%), versus with 1–2 (31% vs. 27%), 3–5 (23% vs. 25%), or ≥ 6 (31% both, *p* = 0.963) people over the recall period.

There was no significant difference between groups for either giving or receiving injecting assistance. Across both groups, peer-to-peer injecting assistance was more commonly performed than received, with 35% of GBMSM and 42% of non-GBMSM reporting having assisted another person’s injection in the past month, compared to 13% of GBMSM and 15% of non-GBMSM reporting having received assistance. Eighty-eight per cent of both GBMSM and non-GBMSM reported no past-month receptive syringe sharing (*p* = 0.531). Of the remaining men, five GBMSM (10%) reported reusing someone else’s used syringe 1–2 times, none (0%) reported doing so 3–5 times, and < 5 (< 5%) reported doing so six times or more (compared to 31 (7%), 10 (2%) and seven (2%) of non-GBMSM, respectively, *p* = 0.531).

#### Health care access

There was no significant difference between groups for accessing drug-related care. Of the whole sample, forty-four per cent of GBMSM and 50% of non-GBMSM reported currently being engaged in any drug treatment (*p* = 0.449). Among participants who only completed a baseline survey (*n* = 159), 88% per cent of GBMSM and 82% of non-GBMSM reported ever having received any drug treatment (*p* = 0.739). Of those who completed a follow-up survey (*n* = 366), 24 GBSM (77%) and 225 non-GBMSM (67%) reported receiving drug treatment in the past 12 months/since last seen (*p* = 0.250). NSPs were reported as the main source of injecting equipment by over 70% of men in both groups (*p* = 0.876). Most GBMSM and non-GBMSM reported not having visited injecting-specific community health clinics for reasons other than to access the NSP in the past 12 months (77% vs. 72%, respectively, *p* = 0.401).

## Discussion

We investigated whether factors previously highlighted as important for understanding injecting drug use among GBMSM (e.g. methamphetamine use, social injecting, and peer-to-peer injecting) translate into quantifiable differences between men stratified by sexuality in a cohort of men who inject drugs in Melbourne. In-line with previous research findings, a larger proportion of GBMSM reported preferring methamphetamine [9, 17, 19, 20, 25] but the related identifiable difference in proportion reporting methamphetamine as their most injected drug was not statistically significant. In this study, the majority of GBMSM (58%) and non-GBMSM (66%) groups reported heroin as their main injecting drug of choice and drug most injected in past month. The predominance of heroin injecting among GBMSM in our study contrasts with findings from studies of drug use in other Australian gay community samples [31], and findings from a similar study conducted in San Francisco by Artenie et al. [17], which both identified methamphetamine as the primary drug injected by men who have sex with men.

Beyond a higher percentage of GBMSM reporting recent HCV testing there was little difference in demographics, drug use, or health service access between the groups. GBMSM were slightly younger than non-GBMSM but had similar socio-economic profiles indicating social disadvantage, which may be the result of street-based recruitment methods. Their socio-economic profiles are comparable to the injecting cohort in San Francisco comprising of men reached through affiliation with people who inject drugs [17], except that in their cohort current homelessness was lower among sexual minority men compared to other men.

Injecting was reported as a similarly social practice independent of sexuality, with just over 1% of men reporting having no connection to other people who inject drugs, and only 14% of men reporting always injecting alone over the past six months. There was evidence of supportive practices within these social networks, with over a third of GBMSM and non-GBMSM reported providing injecting assistance in the past month, and low prevalence of receptive syringe sharing indicating strong adoption of harm reduction practices.

Taken together, these findings suggest that while injecting environments (sexual vs. non-sexual) and social contexts/meanings (e.g. motivations around social connectedness/belonging) may provide a qualitatively distinct experience for sexual minority men in general [26, 34, 39] the actual practice of injecting involves the same material realities [27]. Nonetheless, the higher preference for methamphetamine among GBMSM compared to other men suggests that different approaches to addressing drug-related matters of concern may be required, given its implications for varying risks of harms and differences in treatment availability [40–43]. Moreover, methamphetamine use among GBMSM has previously been associated with sexualised drug use [31, 34] and additional measures may be required to moderate methamphetamine-related risk in these contexts.

In this study, a high percentage of GBMSM reported having ‘ever’ accessed drug treatment at baseline (88%), or having accessed treatment in the past 12 months during follow-up (77%). This is noteworthy, because experiences of sexuality-related stigma in health care settings have been found to contribute to health care avoidance among GBMSM in Australia [44] and few methamphetamine treatment options exist [45]. In comparison, a Canadian study with people who inject drugs in Vancouver found that men who had sex with men accessed treatment less than other men, and they also more commonly reported being unable to access drug treatment in the past 6 months [18]. Meanwhile, in San Francisco there was no evidence for sexuality-based differences in drug treatment access between men who inject drugs recruited through street-based outreach, with low overall treatment access in the past year (~ 30%) [17]. In our study, similar percentages of GBMSM (44%) and non-GBMSM (50%) reported currently receiving any drug treatment. The disparity between our findings and theirs may be influenced by distinctions in health and social security systems, given the many barriers to drug treatment access in the USA [46]. Differences in drug supply and practices between settings may also be impacting on drug treatment availability and access. Methamphetamine has recently overtaken heroin as the most commonly reported last injected drug in Australia [47, 48] and drug treatment services may need to respond to this shift. To date, there is little evidence that fentanyl has penetrated the Australian drug market [47, 49] as it has overseas [40, 50], but concomitant use of methamphetamine and opioids is common in both settings [51–53].

Across both groups, participants primarily obtained injecting equipment through NSPs. Despite typically being co-located with injecting-specific primary health care services in Melbourne, less than a third of participants in either group reported having visited these health care services. Such services are set up with people who inject drugs in mind and should offer low-threshold non-judgmental service provision. This low uptake could point to missed opportunities to engage individuals who might benefit from injecting-specific care related to vein health or prevention and treatment of sexually transmissible infections and blood-borne viruses. For example, in addition to providing linkage to HCV care, these services could be important in increasing awareness of pre-exposure prophylaxis for HIV, which has been found to be low among people who inject drugs in other settings, including among GBMSM [54].

While GBMSM in Australia have a higher risk of HIV acquisition due to higher prevalence and sexual transmission compared to non-GBMSM [55], we found that both groups accessed HIV testing at similar rates. Our study used self-reported data and was unable to determine overall HIV antibody prevalence, however, among those reporting testing for HIV in the past 12 months/since last seen the prevalence was < 5%, with no differences by GBMSM status. This is comparable with HIV antibody prevalence among people who inject drugs in Australian annual surveillance surveys, ranging from 1.5 to 2.5% between 2017 and 2021 [55]. However, HIV antibody prevalence among people who inject drugs was consistently highest among gay men, where it ranged from 20 to 45% over this period, and elevated among bisexual men, where the highest point prevalence of 14% was obtained in 2019. HIV prevalence was also higher among men who reported methamphetamine versus heroin as last drug injected (range: 2.6–4.8%) [55, 56].

The lower HIV prevalence among GBMSM reported in our study may be because GBMSM living with HIV and aware of their HIV status may not have recently tested for HIV and may therefore not be included in this prevalence estimate. An underlying higher HIV prevalence among GBMSM in this cohort could also explain the significantly higher HCV testing rates among GBMSM compared to non-GBMSM (69% vs. 52%, *p* = 0.028), because regular HCV testing is standard care for people living with HIV in Australia [57, 58].

Our findings demonstrate that men who inject drugs in Melbourne continue to be affected by blood-borne viruses and we identified that around 1 in 10 men of both GBMSM and non-GBMSM groups reported reusing somebody else’s syringe in the past month. Overall, these results underscore the importance of promoting free and co-located HIV and HCV testing at NSPs and continued distribution of free needles, syringes and other injecting equipment. We have identified a potentially important subgroup of GBMSM in this Melbourne cohort who may not feel comfortable discussing their drug practices in sexual health services, given the broader stigma attached to injecting [2]. Health care workers should take care to discuss injecting and sexual practices without making their clients feel stigmatised or ashamed about engaging in risky behaviours [47, 54] and avoid shortcuts in addressing their client’s assumed needs based on a salient ‘characteristic’—be it sexuality, drug practice or something else.

Injecting partnerships/networks may be a source of HIV or HCV risk, and our findings regarding social injecting practices demonstrate that these sources of risk exist in equal measure across sexual identities and practices. However, injecting networks also offer protection against risks by providing overdose response, exchanging information on harm reduction, and providing social support [59]. Understanding and moderating the factors contributing to receptive syringe sharing should be a priority, including whether these factors vary by sexuality and may require differentially tailored responses. Exploring such risk and protective factors in our cohort would require additional measures; our questionnaire was not designed to explore social network structures, unlike those used in other studies [59].

Furthermore, our findings raise questions about how recruitment strategies may be missing particular social groups who may objectively be attributable to the same category (e.g. GBMSM who inject drugs) but may not be reachable through sampling strategies targeting GBMSM or people who inject drugs separately. This has obvious implications for how public health perceptions of these groups are shaped [60]. Current GBMSM studies may be failing to represent men who are more socially marginalised (as indicated by the socio-economic profiles of the men in our study), while injecting cohorts may exclude members of socio-sexual networks in which GBMSM inject drugs [22, 34].

Our findings must be considered in light of our small sample size. The proportion of bisexual men in our cohort is greater than in comparable cohort analyses from North America [17, 18]. Their profiles and practices may differ from gay men, but the small sample size prevented us from exploring potential subgroup differences based on sexual orientation/identity (gay, bisexual, queer, heterosexual) and sexual behaviours (having sex with men). Participants were recruited from NSPs and open street drug markets, and may not be representative of GBMSM who inject drugs in Melbourne [31] sourcing their drugs and equipment elsewhere. This study was conducted in metropolitan Melbourne, and it is possible that the social ecologies operating in this setting (e.g. drug market characteristics, public policy, network structures, and sociocultural factors) shaped drug practices in unique ways, hindering generalisation. Finally, self-reported variables are invariably subject to social desirability and recall bias. Nonetheless, discrepancies are generally found to be low among people who use drugs, indicating that their self-reports are sufficiently reliable and valid to provide relevant descriptions of drug practices [61, 62].

## Conclusion

Our findings suggest that except for a greater preference for methamphetamine and higher rates of recent HCV testing among GBMSM, men who inject drugs in Melbourne, Australia, have similar socio-demographics, drug practices and care-seeking behaviours. Hence, this study found little evidence to corroborate the common assumption that sexuality-based differences necessarily equate to variations in social practices, including injection drug use and accessing of care. The higher proportion of GBMSM preferencing methamphetamine suggests that this group may require different responses to their injecting-related health concerns compared to non-GBMSM. However, care must be taken to investigate clients’ needs carefully, beyond relying on assumed knowledge based on salient markers of sexual and social identity. Overall, our study underscores an ongoing need for measures to be taken that address the broader issues of social inequity apparent in this cohort.

## Data Availability

The data analysed during the current study are not publicly available but are available from the corresponding author on reasonable request. Data access requires permission from the Chief Investigator for the cohort (PD).

## Abbreviations

NSP: Needle and syringe program
HCV: Hepatitis C virus
GBMSM: Gay, bisexual and other men who have sex with men
